# Optimizing Yeast Surface-Displayed Unspecific Peroxygenase Production for Sustainable Biocatalysis

**DOI:** 10.3390/bioengineering12080822

**Published:** 2025-07-30

**Authors:** Niklas Teetz, Luc Zuhse, Dirk Holtmann

**Affiliations:** Process Engineering in Life Sciences 2—Electro Biotechnology, Karlsruhe Institute of Technology, Fritz-Haber-Weg 4, 76131 Karlsruhe, Germany; niklas.teetz@kit.edu (N.T.); luc.zuhse@hhu.de (L.Z.)

**Keywords:** unspecific peroxygenases, yeast surface display, enzyme production, production optimization

## Abstract

Unspecific peroxygenases (UPOs) are promising biocatalysts for oxyfunctionalizations in future sustainable economies and can be efficiently immobilized on the cell surface of their heterologous production yeast. This immobilization has versatile uses, ranging from the mL to m^3^ scale; but the production of the yeast surface displayed UPOs, and their handling has yet to be optimized to advance sustainable industrial processes in light of the UN’s sustainable development goals. Here, we present optimized production protocols for surface-displayed UPOs for shaken and stirred systems in different scales and describe suitable storage conditions and a sterilization method. We utilized one-factor-at-a-time and design of experiments approaches. We were able to streamline published protocols for shaken flask cultivations to achieve a 60% increase in volumetric activity, using reduced amounts of media. We also show at least a doubling of final activity for bioreactor cultivations by utilizing a different medium than the industry standard. Finally, we present a novel, robust protocol for parallelized methanol-induced enzyme production in *Komagataella phaffii* in a BioLector XT^®^ reactor. Enzyme activity did not decrease and even increased by our recommended sterilization method and during storage over 87 days. This study aims to advance the yeast surface display immobilization method by providing methods for efficient production, storage and utilization of this promising biocatalyst.

## 1. Introduction

Unspecific peroxygenases (UPOs) are emerging biocatalysts with promising properties for application in industrial catalysis due to their ability to regio- and stereoselectively hydroxylate non-activated C-H bonds in a variety of substrates, utilizing hydrogen peroxide as the only cofactor [1,2,3,4,5]. These reactions are rare in conventional organic chemistry, underlining the potential of enzymatic catalysis with UPOs in a future circular economy. Utilization of biocatalysts can contribute to achieving sustainable development goals (SDGs), like the ones set by the United Nations (UN) [6]. In particular, UPO-based processes have the potential to replace a number of conventional petrochemical production processes due to their substrate promiscuity [3,7,8,9]. Therefore, they will directly impact SDG 13 (climate action) by reducing the emitted CO_2_ equivalents of those processes but also SDGs 8 (decent work and economic growth) and 9 (industry, innovation and infrastructure) through the innovative transformation of industry branches, securing economic growth for future businesses that will likely face high prices for emitted greenhouse gases due to political and societal pressure. Furthermore, the biobased production and biodegradability of enzymes such as UPOs contribute to SDG 12 (responsible consumption and production) by reducing waste and pollution and therefore fostering circular economy development and sustainable production practices. One of the weak points of UPO-catalysis is the low activity yields of conventional immobilization procedures (≤10%) [10,11], reducing the space time activity (STA; units of immobilized enzyme per litre and day of fermentation; [12]) for the biocatalyst production drastically. We recently published an immobilization approach via a yeast surface display that showed much better recovered activity efficiency (RAE), STA and E Factor (for the catalyst production) [12] than conventional immobilization approaches [10]. A comparison of the single phases of immobilized UPO production has, however, shown that these advantages stem purely from the immobilization itself, while the fermentation procedure was less effective than for the production of free UPO [12]. We mainly attribute this to the differences between producing free, secreted UPO and a yeast surface displayed UPO, where the attachment points for immobilization are limited by the available cell wall, the fact that attachment might be more efficient during growth (creation of new cell wall) than on old cell wall material and different environmental requirements (mainly temperature) for the correct folding of UPO with attached anchor protein to the free UPO. Therefore, we aimed to optimize production specifically for these surface displayed UPOs by adaption of existing protocols for the production of free UPO.

Additionally, YSD has potential for easy screening of immobilized UPO variants, which has not yet been explored. Advantages are the non- or minimal purification needed for high-purity enzymes, as well as the simple concentration of the enzymes without the necessity for ultrafiltration devices [12]. Furthermore, YSD-UPO variants can be screened in the already immobilized state, so screening results take into account the effects of the enzyme variants’ immobilization. With conventional methods, immobilization effects are either ignored during screening and can later be detrimental to enzyme activities or the immobilization procedure has to be applied to all produced variants prior to screening. One of the downsides of YSD as a screening tool is the inevitable presence of cells in the screening assays, which can interfere with certain measurement methods like photometry. This interference can be minimized by optimizing the enzyme activity per cell, reducing the number of cells necessary in the assay.

In this study, we present optimized production protocols for YSD-UPOs, ranging from 1 to 2 mL scale in a BioLector XT^®^ for screening applications, to shaking flask scale for small-scale lab production, and up to a 5 L bioreactor for larger-scale lab production and as a starting point for scale-up for pilot and industry scale. Furthermore, we present a simple method for sterilizing the cells used as an immobilization matrix without compromising enzyme activity (or causing enzyme bleeding). We hope these optimized protocols help establish yeast surface display as a cheap and easy immobilization method for UPOs and as a screening tool for UPO research.

## 2. Materials and Methods

### 2.1. Cultivation of YSD-UPO-Producing Komagataella phaffii in Shaking Flasks

A total of 3 mL of YPD medium (20 g L^−1^ peptone, 20 g L^−1^ dextrose, 10 g L^−1^ yeast extract) with 100 µg mL^−1^ zeocin was inoculated from a cryoculture of *Komagataella phaffii* (also known as *Pichia pastoris*) X33 pPpB1_*Aae*UPO_PaDa-I_SAG1 [10] and incubated in a cultivation tube at 30 °C, 180 rpm, at a 45° angle. After 48 h, 25 mL of BMGY medium (100 mM KP_i_ pH 6, 10 g L^−1^ yeast extract, 20 g L^−1^ peptone, 10 g L^−1^ glycerol, 400 µg L^−1^ biotin, 3.2 mM MgSO_4_, 1.7 g L^−1^ yeast nitrogen base without amino acids, 10 g L^−1^ (NH_4_)_2_SO_4_)) with 100 µg mL^−1^ zeocin in a 500 mL baffled shake flask were inoculated from the YPD cultures to an OD_600_ of 1. The BMGY cultures were then incubated at 30 °C at 180 rpm for 45 h (OD_600_ ≈ 20) before the entire culture was transferred to a 50 mL Falcon tube and centrifuged at 4 °C, 4816× *g* for 15 min. The supernatants were discarded and cells were resuspended in 12.5 mL BMMY medium (100 mM KP_i_ pH 6, 10 g L^−1^ yeast extract, 20 g L^−1^ peptone, 5 g L^−1^ methanol, 400 µg L^−1^ biotin, 3.2 mM MgSO_4_, 1.7 g L^−1^ yeast nitrogen base without amino acids, 10 g L^−1^ (NH_4_)_2_SO_4_)) with 100 µg mL^−1^ zeocin each (OD_600_ ≈ 30) and transferred back into baffled shake flasks. The cultures were incubated at 30 °C or 25 °C, 180 rpm. After 4 h of methanol acclimatization, a regular methanol feed of 1% (*v*/*v*) per day was started (0.67% (*v*/*v*) in the afternoon and 0.33% (*v*/*v*) in the morning). OD_600_ and ABTS activity were routinely measured over the fermentation duration.

#### 2.1.1. Temperature During Protein Expression

A total of 6 × 3 mL of YPD were inoculated, incubated and used to inoculate 6 main cultures in BMGY as described above. Upon medium change to BMMY, 3 of the cultures were incubated at 30 °C and 3 were moved to incubation at 25 °C.

#### 2.1.2. Cultivation Volume Reduction

For investigation of the impact of the cultivation volume after medium exchange, six flasks with 25 mL BMGY medium were inoculated and incubated as described above, and the medium was changed to BMMY after 45 h. For three of the flasks, the entire culture was centrifuged and resuspended in 25 mL BMMY. For the other three, the volume was reduced by resuspending in 12.5 mL, and all six cultures were incubated at 25 °C with methanol feeding as described above.

#### 2.1.3. Induction at Different Optical Densities

To determine the impact of the optical density at MeOH induction on volumetric activity, 4 shake flasks were inoculated and incubated as described above, and the medium was changed to BMMY at different optical densities. In other experiments, induction was conducted at OD_600_ of around 20, so we changed the medium at OD_600_ of 14, 20, 25 and 29 in this experiment. After induction, the cultures were incubated for another 75 h, and optical density and volumetric activity were measured regularly.

### 2.2. Cultivation of YSD-UPO-Producing Komagataella phaffii in a Bioreactor

Bioreactor cultivations were conducted in a 5 L bioreactor (Biostat B5, Sartorius AG, Goettingen, Germany), equipped with two Rushton turbines. A total of 3 mL YPD medium with 100 µg mL^−1^ zeocin was inoculated from a *K. phaffii* strain X33_pPpB1_*Aae*UPO_PaDa-I_SAG1 [10] cryoculture and incubated at 30 °C, 180 rpm in a cultivation tube at a 45° angle for 48 h. The YPD culture was then used to inoculate 4 × 50 mL BMGY medium with 100 µg mL^−1^ zeocin in four 500 mL baffled shaking flasks. The BMGY cultures were incubated at 30 °C, 180 rpm for 16 h. The four BMGY cultures were then used to inoculate 2 L basal salt medium or BMGY, both with 12 g L^−1^ Pichia Trace Metal solution 1 (PTM_1_) [13] in the bioreactor (to OD ≈ 1) with pH adjusted to 5. Temperature during the fermentation was initially set to 30 °C with an aeration rate of 4 L min^−1^ and a 400 rpm stirrer speed. After the glycerol batch phase ended, indicated by a rapid increase in DO, a glycerol feed was started until an optical density of OD_600_ ≈ 150 was reached. Subsequently, the temperature was set to 25 °C, and a methanol feed with 12 g L^−1^ PTM_1_ was started. Aeration and stirrer speed were gradually increased to keep DO above 20%. Contraspum A4050 was added when necessary to control excessive foaming (<1 mL total). Optical density and volumetric enzyme activity were measured regularly (at least 3 times daily and at every process phase transition). Fermentation was stopped when volumetric activity did not increase over the course of >3 measurements (basal salts medium) or when the reactor volume was limiting further feeding (BMGY medium).

### 2.3. Optimization of Cultivation Parameters in BioLector XT^®^ Cultivations and Design of Experiments

The Software Design Expert^®^ (version 23.1.1 64-bit, Stat-Ease, Inc., Minneapolis, MN, USA, “www.statease.com (accessed on 17th June 2025)”) was used for the design of the experiments, and a custom response surface design was applied. The parameters were as follows: A: glycerol in the medium (0%, using BMMY medium with 0.5% methanol instead—3%), B: inoculation OD_600_ (0.1–5), C: feed volume of methanol per pulse during methanol fed batch phase (0.5–27 µL/0.06–3% (*v*/*v*)) and D: cultivation temperature (22 °C, 27 °C or 32 °C) were varied for optimization. The optimized responses were maximally reached volumetric enzyme activity and maximally reached enzyme activity per cell density. The cultivations were conducted in a parallelized microbioreactor BioLector XT^®^ with microfluidics module (Beckman Coulter Life Sciences, Indianapolis, IN, USA, “www.beckman.com/microbioreactor/biolector-xt (accessed on 17 June 2025)”).

The design featured 19 runs to build the model and was supported by 7 lack of fit runs, 7 replicate runs and 4 center point runs. Of the 39 total runs, 9 runs led to no successful cultivation (5 model runs, 2 lack of fit runs, 1 replicate run and 1 centre point run) due to either an incompatibility of the parameters with the biological requirement of the cells, reproducibility issues in the BioLector XT^®^ or human inconsistency in the preparation of the BioLector plates. Across the varied parameters, the unsuccessful runs occur without a noticeable pattern, except that there was no failed run among the cultivations that started with 0% glycerol. Since the design was built quite robust, the failed runs could be ignored and the predicted model was still significant.

### 2.4. Enzyme Activity Assays

The enzymatic activity was routinely measured by the oxidation of 2,2′-azino-bis(3ethylbenzothiazoline-6-sulfonic acid) (ABTS) to a stable radical. For the assay, 100 µL 3 mM ABTS, 10–100 µL of sample cell suspension (to an optical density of ≈0.5) and 50 µL of 40 mM H_2_O_2_ were added in this order to the McIlvane buffer of pH = 4.5 to a total volume of 1 mL. Absorbance at 420 nm was measured and activity calculated via the Beer–Lambert law with an extinction coefficient of ABTS ε_420_ = 36,000 M^−1^ cm^−1^.

## 3. Results

### 3.1. Cultivation of YSD-UPO-Producing Komagataella phaffii in Shaking Flasks

Cultivation in shaking flasks was the first published method for the production of YSD-UPOs and is likely the simplest and commonly used method in academic research. Methanol-induced expression in *Komagataella phaffii* in shaking flasks is generally divided into a biomass-generating glycerol batch phase and an expression-inducing methanol acclimatization and subsequent fed batch phase. Since the used *AOX*1 promoter is inhibited by glycerol and online analysis of the amount of glycerol in the medium requires special equipment, the cells are routinely centrifuged after the glycerol batch phase and resuspended in fresh medium containing methanol instead of glycerol. Some studies use half the amount of methanol-containing medium for resuspension compared to the amount of glycerol-containing medium, likely to improve oxygen input into the medium when using baffled shake flasks [14,15]. Alternatively, to the medium exchange, it is possible to measure the cell growth and introduce methanol into the medium after the culture reaches the stationary phase, but this strategy necessarily leads to a starvation phase of the culture before protein expression, which can influence performance. Both protocols can be viewed in a previous publication on the matter [10].

We had reliable results with the method, including medium change, and decided to investigate three main cultivation parameters: temperature during protein expression, optical density at medium change and the volume of methanol medium after medium change for activity optimization.

#### 3.1.1. Temperature During Protein Expression

**Figure 1:**
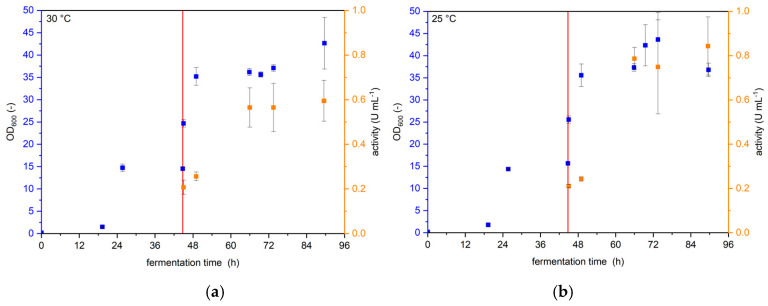
Cultivation of *K. p.* pPpB1_PaDa-I_SAG1 with constant temperature or reduced temperature after medium change. The red line indicates the medium change. Values are given as average with standard deviation (*n* = 3) (**a**) optical density and volumetric ABTS activity with constant temperature of 30 °C (**b**) optical density and volumetric ABTS activity with cultivation at 30 °C until medium change and subsequent cultivation at 25 °C.

The cultivation temperature has a major impact on the expression of proteins. Higher temperatures generally lead to faster transcription and translation due to the RNA polymerase and ribosome activity following the Arrhenius law. However, the fast production of the enzyme’s primary amino acid chain can hinder the correct folding of the enzyme and therefore reduce the fraction of functional enzymes drastically. Additionally, post-translational modifications that are directly linked to the enzymes’ activity or stability can sometimes be conducted incompletely at higher temperatures if the enzymes are secreted out of the cell or misfolded. Furthermore, oxygen solubility in aqueous media is increased at lower temperatures, which is especially important for expression in oxygen-demanding host organisms like yeasts. Therefore, protein expression, especially in heterologous expression hosts, such as in yeasts, is often conducted at lower temperatures than the organism’s optimal growth temperature. The Invitrogen Pichia Fermentation Process Guidelines [13] suggest lowering the temperature to 25 °C during methanol phases, and the expression of free UPOs in *Komagataella phaffii* has routinely been conducted with this temperature shift [16,17]. Here, we investigated whether the production of YSD-UPOs is improved if the temperature is reduced from 30 °C during biomass growth to 25 °C during protein expression, over leaving the temperature at 30 °C. The results are shown in Figure 1.

Although the maximal OD_600_ reached around 43 h for both cultivation temperatures, volumetric activity was 42% higher after 90 h when cultivating at 25 °C (0.84 U mL^−1^) than when cultivating at 30 °C (0.59 U ml^−1^). All following cultivations in shaking flasks were performed with the temperature reduction to 25 °C upon medium exchange.

#### 3.1.2. Cultivation Volume Reduction

**Figure 2:**
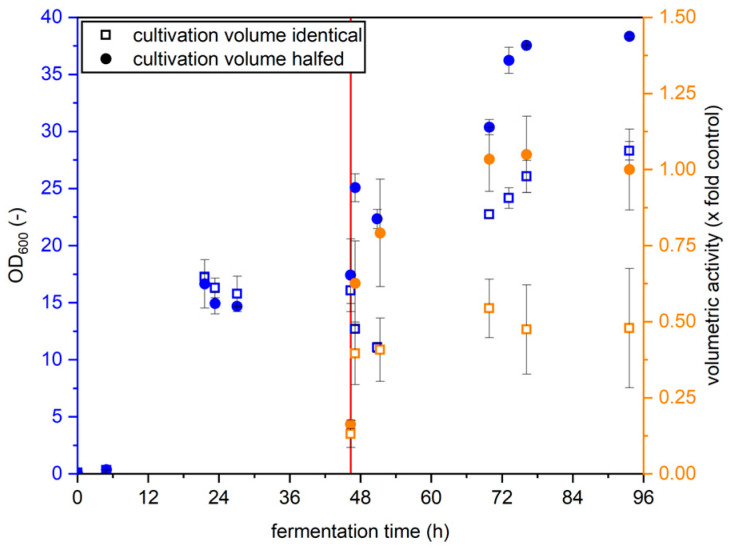
Comparison of OD_600_ and volumetric activity of *K. p.* pPpB1_PaDa-I_SAG1 cultivations in shaking flasks with resuspension in BMMY of a volume identical to BMGY at medium exchange (squares) or halved volume (circles). The red line indicates the time of medium exchange. Values are given as average with standard deviation (*n* = 3).

For the production of yeast surface displayed enzymes with *Komagataella* under methanol induction, the cultivation volume is routinely halved after medium exchange. Here, we investigated the influence of halving the cultivation volume on the production of YSD-UPOs. The results are shown in Figure 2.

The total activity produced was identical for both cultivation volumes after two days of methanol feed, and volumetric activity was therefore double when cultivation volume was halved. Final optical density was more than half when leaving the cultivation volume identical compared to when halving it, so the activity per cell is higher when halving the cultivation volume. For cultivations much longer than this, it is feasible that methanol is eventually no longer the limiting substance in the medium, giving the larger resuspension volume an advantage over halving the volume. However, since this effect was not observed for experiments within this study, we halved the volume upon resuspension to conserve medium components.

#### 3.1.3. Induction at Different Optical Densities

**Figure 3:**
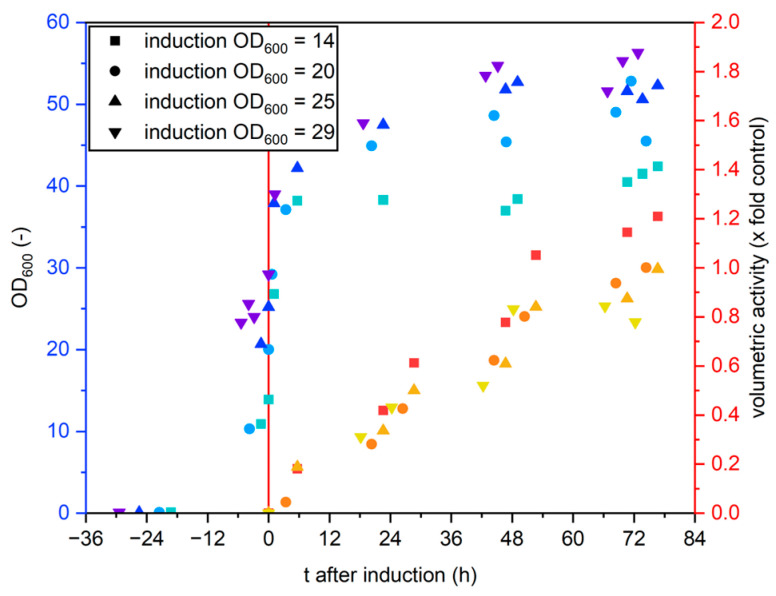
Cultivation of *K. p.* pPpB1_PaDa-I_SAG1 with medium exchange at different optical densities. Optical density (blue–teal colors) and volumetric activity (red–yellow colors) are plotted against the time after induction. The red line indicates the medium change and the x-axis is scaled, so the time of induction (=medium exchange) is t = 0.

We investigated the impact of the OD_600_ at medium exchange on the final volumetric activity as well as the activity per cell by exchanging the medium at different OD_600_ values. The results are summarized in Figure 3.

Both the final volumetric activity as well as the volumetric activity per OD_600_ were highest for induction at OD_600_ = 14 and lowest for OD_600_ = 29, indicating that the induction should be performed at low optical density.

### 3.2. Comparison of Protocols for YSD-UPO Production in a Bioreactor

Yeast-based production of enzymes is typically performed in stirred reactor systems (commonly in stirred tank reactors, STRs) from benchtop scale to multi-cubic meter industrial scale. To showcase the production of YSD UPOs in a stirred instead of shaken system, we cultivated the Yeast strain *Komagataella phaffii* X33 pPpB1_PaDa-I_SAG1 [10] in a bioreactor, following two different protocols, and measuring process parameters to identify the better protocol.

**Figure 4:**
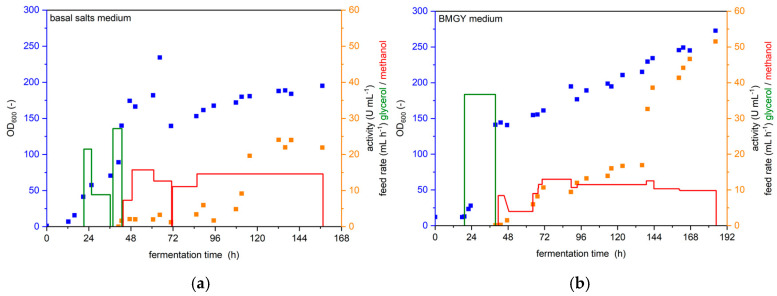
Cultivation of *K. p.* X33 pPpB1_PaDa-I_SAG1 in a 5 L bioreactor. The optical density (blue), volumetric activity (orange), glycerol feed rate (green) and methanol feed rate (red) are shown over the duration of the entire cultivation. (**a**) cultivation with basal salts medium (**b**) cultivation with BMGY medium.

In the literature, UPO production with *Komagataella phaffii* is typically either performed in a minimal medium, like basal salts medium, in a stirred reactor [13,17,18,19,20] or a complex medium like BMGY medium in shaking flasks [20,21,22]. However, Tonin and coworkers have conducted a pilot-scale fermentation of *K. phaffii* for the production of a variant of the UPO from *Agrocybe aegerita*, *Aae*UPO PaDa-I, using BMGY medium in a stirred tank reactor [16]. Here, we compare cultivation with both media in a 5 L bioreactor and measure the volumetric activity. We compared the 5 L cultivation with BMGY medium to the cultivation of Tonin and coworkers in a previous publication [12]. For the first reactor run, we followed the Invitrogen fermentation guidelines for *Pichia pastoris* and used a basal salts medium [13] with 4% glycerol. For the second reactor run, we followed the protocol of Tonin and coworkers [16] and used the complex BMGY medium with 1% glycerol. The results for both runs are shown in Figure 4.

The production of UPOs was successful utilizing both media. However, cultivation in BMGY medium leads to drastically improved volumetric activity compared to the basal salt medium. Maximal activity was 22–24 U mL^−1^ after 132 h of cultivation in basal salts medium, but did not increase further within the next 24 h, so cultivation was stopped after 157 h. At 132 h, volumetric activity was slightly lower in the BMGY medium run, at 17 U mL^−1^. However, activity increased rapidly afterwards to 41 U mL^−1^ at 160 h and 51 U mL^−1^ at 184 h, at which point the cultivation was stopped because the reactor volume did not support further feeding, although activity was still increasing at this time.

Complex media complicate thorough balancing of the cultivation process due to the usually unknown nature of some of their components, like yeast extract and peptone. Additionally, complex media are typically more expensive per litre than minimal media, which has to be considered especially in large-scale fermentations. However, if the goal of the cultivation is to maximize YSD-UPO production, the complex BMGY medium is recommended over the minimal basal salts medium, so we decided to use BMGY in our optimization of YSD-UPO production in a BioLector XT^®^.

### 3.3. Optimization of YSD-UPO Production in a Parallelized Microfermentation System—BioLector XT^®^

Yeast surface display has the potential to be a valuable tool for the screening of new UPOs or UPO variants. The simple downstream processing by centrifugation enables a purification of the displayed enzyme, and if the enzyme is intended to be immobilized in its final use case, the screening can easily be performed in an immobilized state. A downside is the difficult determination of production levels of the protein because of its attachment to the cells. However, a proper determination of production levels usually requires purification for non-surface displayed enzymes as well. A high production activity is desirable in any case, as it minimizes the required amount of sample as well as the assays needed for sensitivity. The parallelized microfermentation reactor BioLector XT^®^ enables versatile process control for the cultivation on the 1–2 mL scale and production of enzymes or other substances of interest. Here, we optimized the cultivation process for YSD-UPOs in a BioLector XT^®^ to advance this tool’s applicability for the screening of UPO variants.

For this optimization, we conducted a design of experiments to determine optimal production conditions in BioLector FlowerPlates^®^. Since sampling of the cultivation wells requires pausing the BioLector XT^®^ protocol, but we wanted to optimize the volumetric UPO activity, we determined UPO expression by using a YSD-UPO strain with sfGfp fused to the UPO PaDa-I [10], measuring sfGfp fluorescence. However, expression and activity are not necessarily proportional to each other, especially in heterologous enzyme production, since a higher temperature might lead to a faster protein biosynthesis rate but simultaneously reduce the portion of correctly folded protein before its secretion. Therefore, we correlated sfGfp- fluorescence and UPO activity (specifically ABTS-activity) at 22 °C, 27 °C and 32 °C by cultivating the same strain that we used in the BioLector XT^®^ cultivations in shaking flasks, measuring optical density and activity at four different time points, but also transferring a part of the culture to a BioLector XT^®^ plate and immediately measuring biomass by backscatter and sfGfp fluorescence. As a control, we cultivated and measured a strain without the sfGfp fused to the UPO under identical conditions.

#### 3.3.1. UPO Activity/sfGfp Fluorescence Correlation

Indeed, we found that the correlation between sfGfp-fluorescence and UPO activity is dependent on the cultivation temperature. Additionally, we found a correlation between sfGfp fluorescence and UPO activity not only for the sfGfp-strain but also for the control strain at the temperatures 22 °C and 27 °C, which was not expected. sfGfp fluorescence also correlated to the backscatter signal, which was expected for the sfGfp-strain, but, again, not expected for the control strain. Since the BioLector XT^®^ measures both backscatter and sfGfp, we were able to correct the sfGfp fluorescence by the backscatter signal using the sfGfp/backscatter correlation of the control strain. The corrected Gfp signal subsequently showed no more correlation to the UPO activity of the control strain, but still good correlation to the activity of sfGfp strain.

Additionally, the slope and intercept of the linear regressions were linearly dependent on the temperature of the cultivation, allowing for activity calculations from sfGfp-fluorescence at temperatures not characterized.

The OD_600_ was shown to be linearly dependent on the backscatter signal in the observed range, and the correlation, like the UPO activity/sf-Gfp fluorescence correlation, is dependent on the cultivation temperature. All correlations are shown in the Supplementary Figures S1–S7.

These results allowed us to continuously determine OD_600_ and UPO activity inside BioLector FlowerPlates^®^ without pausing the cultivation protocol and drawing samples by monitoring sfGfp fluorescence and backscatter.

#### 3.3.2. Design of Experiments

**Figure 5:**
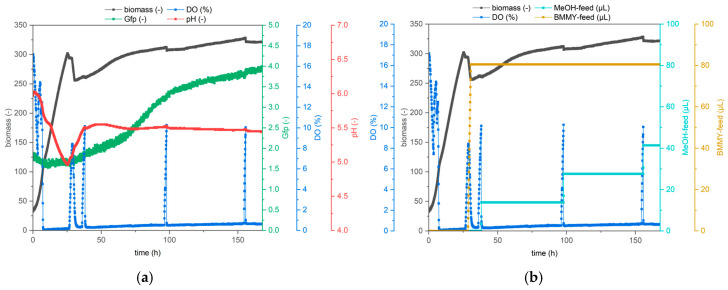
Example cultivation progress of methanol-induced YSD-UPO production in a BioLector XT^®^. Biomass, measured via backscatter signal, is shown in black and dissolved oxygen in blue. For a clear overview, only the first 168 h are depicted; (**a**) pH value development (red) and Gfp signal development (green); (**b**) automated feeding profiles of BBMY (yellow) and methanol (light blue).

To find the optimized YSD-UPO production protocol for BioLector XT^®^ cultivations, the Invitrogen fermentation guidelines for *Pichia pastoris* were used as general template for the BioLector XT^®^ protocol: a glycerol batch phase followed by a glycerol fed batch phase, a methanol batch phase and a methanol fed batch phase, using dissolved oxygen (DO) as an indicator for the end of the batch phases and to dose the feed during fed batch phases. Since the BioLector XT^®^ microfluidic FlowerPlates^®^ feature two reservoir wells for feed solutions, we had to decide between glycerol, methanol and pH-regulation solutions. Since the glycerol-fed batch phase is used to increase biomass, we decided to regulate biomass by using different glycerol amounts in the glycerol batch phase and replacing glycerol as the feed solution. Since the first tests showed that pH regulation was not necessary to achieve measurable activity levels and the transition between C-sources can lead to temporarily erratic DO levels [13], which can lead to overdosing of pure methanol and therefore the death of the culture, we decided to have a BMMY medium reservoir for the methanol batch phase and methanol acclimatization and pure methanol for the subsequent methanol-fed batch phase. Alternatively, methanol acclimatization can be achieved from the same reservoir well as the methanol-fed batch by utilizing a custom LUA script, or if the BioLection software allows for that option, in future versions. Here, we want to offer an immediately usable, optimized solution for BioLector XT^®^ users who have no coding knowledge, so we decided to use both wells for methanol acclimatization and feeding. Furthermore, we chose to apply an aeration rate of 30 mL min^−1^ and a shaking frequency of 1200 min^−1^ to enable high oxygen transfer rates for optimal yeast fermentation. The starting volume was 800 µL, and the upper volume limit was determined by the shaking frequency to be 1400 µL. After the first batch phase, signified by a rapid increase in the DO, 80 µL of BMMY was fed with a feed rate of 80 µL h^−1^ to each well for methanol acclimatization. After that, methanol was completely consumed, signified by another rapid increase in DO. Then, the methanol-fed batch was started. An exemplary cultivation progress along these phases is shown in Figure 5. The cycle time for each run was set to 5 min 30 s, determined by the shortest cycle time that was possible for all experiments (more runs per plate lead to longer cycle times). Cultivation was stopped after 10 days, and the recorded data was evaluated for maximal volumetric activity and maximal volumetric activity per OD_600_.

The evaluated responses were the maximally reached activity and the maximally reached activity per OD_600_.

**Figure 6:**
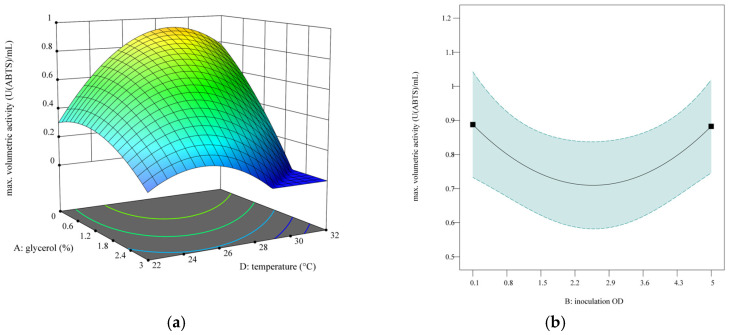
Optimization results for the maximally reached volumetric activity. (**a**) predicted quadratic 2-factor interaction of the glycerol % in the starting BMGY medium and cultivation temperature at inoculation OD_600_ = 0.1. (**b**) predicted quadratic influence of the inoculation OD_600_ on maximally reached volumetric activity at 0% glycerol in the starting BMGY medium and at 27 °C.

**Figure 7:**
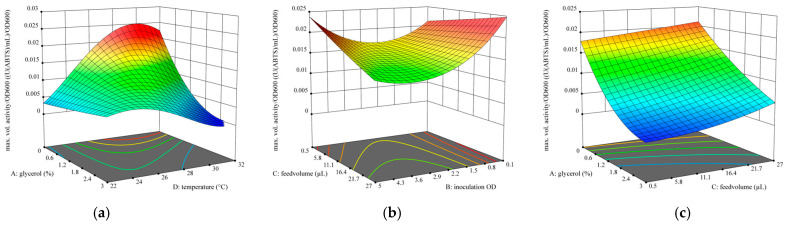
Optimization results for the maximally reached activity per OD_600_. (**a**) predicted quadratic 2-factor interaction of the glycerol % in the starting BMGY medium and cultivation temperature at inoculation OD_600_ = 0.1 and methanol feed volume of 27 µL per pulse. (**b**) predicted quadratic 2-factor interaction of the methanol feed volume and inoculation OD_600_ at 0% glycerol in the starting BMGY medium and cultivation temperature of 29 °C. (**c**) predicted quadratic 2-factor interaction of glycerol % in the starting BMGY medium and methanol feed volume at inoculation OD_600_ = 0.1 and cultivation temperature of 27 °C.

For optimization of maximal activity, the quadratic model was strongly reduced to the terms A, B, D, AD, A^2^, B^2^ and D^2^ due to other terms being not significant. The terms B and D are also not significant (*p*-values of 0.9372 and 0.6146, respectively) but were kept for model hierarchy because the terms B^2^ and D^2^ were significant (*p*-values of 0.0089 and <0.0001, respectively). The full model and analysis of variances (ANOVA) with Lack of Fit for this model and the model for maximally reached activity per OD can be found in the supplementary information (Tables S1–S4). The term C (MeOH feed volume) was not a significant factor for maximally reached activity. A graphic representation of the modeled influence of the parameters A, B and D on maximally reached activity can be found in Figure 6, and results for maximal activity per OD_600_ can be found in Figure 7.

Quadratic dependence of the activity on the glycerol % and temperature (Figure 6a) indicates that directly starting in BMMY (0% glycerol) for methanol acclimatization leads to the best results. This is in agreement with the shaking flask results, which indicate that a lower OD at medium change is beneficial for volumetric activity (chapter 3.1.3). The temperature optimum depends on the amount of glycerol used and is between 29 °C and 25 °C for 0% and 3% glycerol, respectively. According to the model, the impact of inoculation OD (factor B) is independent of other factors, and either minimal or maximal OD is best for maximally reached activity.

For optimization of maximal activity per OD, the quadratic model was reduced to the terms A, B, C, D, AC, AD, BC, B^2^ and D^2^ due to other terms being not significant. The terms C and D are also not significant (*p*-values of 0.1355 and 0.5998, respectively) but were kept for model hierarchy because the terms AC, BC and AD and D^2^ were significant (*p*-values of 0.0153, 0.0277 and <0.0001 and <0.0001, respectively).

The quadratic dependence of the activity per OD on glycerol % and temperature (Figure 7a) is very similar to the dependence of the maximally reached activity, which is not surprising. Starting directly in BMMY (0% glycerol) leads to the highest activity per OD except when the cultivation temperature is 23 °C or less. The temperature optimum for 0% glycerol was slightly higher than in the first model, at around 30 °C. The dependency on feed volume and inoculation OD (Figure 7b) shows that inoculation OD should either be high or low, like for the maximally reached activity, but the optimal MeOH feed volume depends on the inoculation OD. For high inoculation OD, a low feed volume is recommended and vice versa. Interaction between glycerol % and MeOH feed volume (Figure 7c), shown at an inoculation OD of 0.1, reveals that glycerol % has a bigger impact on the activity per OD than the MeOH feed volume.

For confirmation of the model, we predicted the responses for two points and cultivated each point in multiple replicates:

1. Goal: maximize the maximally reached activity with consideration of reaching high activity per OD (six replicates).
  o % glycerol = 0
  o Inoculation OD = 0.1
  o MeOH feed volume = 0.65 µL
  o Temperature = 29.2 °C
2. Goal: good maximally reached activity without consideration of activity per OD and using significantly different parameters than for one (four replicates).
  o % glycerol = 0
  o Inoculation OD = 5
  o MeOH feed volume = 17.2 µL
  o Temperature = 25.6 °C

The model was confirmed for both predicted points and for both the responses (data mean was within two-sided 95% confidence of the prediction, Table S5).

Since the results suggest that starting in BMMY is preferred over any percentage of glycerol in the start medium, the methanol acclimatization happens at the start of the first batch phase and the 80 µL feed of BMMY after the end of the first phase is not necessary. This allows for a simplification of the protocol to two phases: the methanol batch phase and the methanol-fed batch phase, and it frees up one of the feeding wells. Since pH values did not deviate much from the starting pH, this well can be used for custom applications tailored to the specific experiment or can be left empty. We therefore recommend the following protocol for screening experiments with YSD-UPOs in a BioLector XT^®^:

Inoculate BMMY medium from a preculture to an OD_600_ of 0.1 or from a colony to a volume of 800 µL.
Choose your filter modules (DO required).
Cycle time 00:00:00–00:10:00 is recommended.
Humidity control: on.
Temperature: constant 29 °C.
Shaking frequency: constant 1200 rpm.
Air: constant 30 mL/min.
Set the maximum volume of cultivation wells to 1400 µL.
Define feed wells for methanol feed.
Enable signal-triggered methanol feed.
Signal: DO > 15.00; pause: 15 min; pump volume: 0.65 µL (increase pause time for higher pump volumes to avoid accidental multiple feeding).
1. Start condition: DO < 12.0; block 20 min.
2. Start condition: DO > 15.0; block 20 min.

### 3.4. Sterilization and Storage of YSD-UPO

Immobilization of living cells can lead to catalysis-related problems, such as the import, metabolism, or adherence of substrates and products in and on the cells. Furthermore, stress responses could lead to autolysis and change the structure of the immobilization carrier [10]. A sterilization procedure that preserves the activity of the enzymes is therefore desirable. We centrifuged stocks of KP_i_ buffer stored cells (OD > 600), resuspended them in a variety of organic solvents and inverted them for 4 h at room temperature. Afterwards, cells were again centrifuged and resuspended in KP_i_ buffer. A total of 100 µL was then plated on YPD agar plates and incubated at 30 °C for 7 days. After incubation, the colony-forming units were counted. Additionally, ABTS activity was measured and compared to the activity before organic solvent exposure.

A total of 4 h of inversion in isopropanol led to 0 colonies on the agar plates and preserved and even enhanced activity (136% of initial activity) and was therefore deemed the best method for sterilization. This activation of YSD-UPOs by exposure to solvents was previously described and discussed [10], and even the activation of free UPO has been shown [23,24].

To evaluate storage conditions for YSD-UPOs, we used cells from shaking flask cultivations and stored them under different conditions in a 100 mM KP_i_ buffer with pH = 7. The cell suspension was aliquoted and stored at either room temperature (RT), 4 °C, −20 °C or −80 °C because these are the common storage temperatures available in most biological wet labs. To investigate the impact of frequent thawing and refreezing, samples were brought to RT 5 times a week and then moved back to storage conditions. Activities were measured weekly for 12.5 weeks (87 d) in triplicate and after samples were brought to RT. The standard assay temperature of 30 °C was used for the measurement. The results can be seen in Figure S8a. Additionally, the final activity after 87 d was compared to freeze-dried samples stored dry at the four temperatures and for wet-stored samples stored at −20 °C and −80 °C without frequent thawing. Freeze-dried samples were resuspended in 100 mM KP_i_ pH 7 before measurement. The comparison can be seen in Figure S8b.

Based on our results, we can recommend any storage temperature other than RT for the duration of this experiment because we measured an activation effect by the cold storage rather than a deactivation. Storage at −20 °C and −80 °C had the overall best effect on enzyme activity. Frequent freezing and thawing also did not deactivate the UPOs but rather enhanced the activation effect. Freeze-drying YSD-UPOs did lead to small activation for storage at −20 °C and −80 °C and a preservation of activity for storage at 4 °C. For storage at RT, freeze drying did not lead to a significant improvement of activity after 87 d versus wet storage. We suspect that the activation effect can be at least partially attributed to the formation of ice crystals during freezing, which compromises cell integrity. Mechanical destruction of the cell structure could lead to improved diffusion conditions for the immobilized enzymes, causing an apparent activation. The comparatively small activation of freeze-dried samples seems to support this hypothesis, but the strong activation of wet-stored samples at 4 °C contradicts it.

## 4. Discussion

Within this study, we showed optimized production of YSD-UPOs in shaking flasks, bioreactors and the BioLector XT^®^ and presented methods for storage and sterilization of YSD-UPOs.

For cultivation in shaking flasks, we were able to increase volumetric activity by 40% by decreasing the cultivation temperature after methanol induction from 30 °C to 25 °C, and another 20% by starting induction at a lower OD_600_. Changing the cultivation volume upon induction did not influence final volumetric activity.

For bioreactor cultivations, we showed that usage of the complex medium BMGY led to at least a doubling in produced volumetric activity compared to using the minimal medium suggested by the Invitrogen cultivation guidelines for *Pichia* [13]. While the use of complex media might not be suitable for some applications where a precise balancing is necessary, it is recommended for the production of UPOs in *K. phaffii*.

We presented a robust protocol for the cultivation of *Komagataella phaffii* in a BioLector XT^®^ and the methanol-dependent production of proteins, in this case, yeast surface displayed UPOs. For this, we optimized the amount produced and activity per cell in a design of experiments approach.

This study focuses exclusively on process-related optimization. However, because YSD-UPOs are a product of yeast fermentation, the engineering of the yeast strain itself can also improve production. For example, changes in signal peptides or promoters [21,25,26,27], the introduction of linkers between the anchor protein and the protein of interest [28,29], the coexpression of chaperones [30] or reducing proteinase activities in the yeast [31,32] can all influence the productivity of the strain and have partially been explored for the expression of free UPO and other enzymes in *Komagataella* and other yeasts, but not for YSD-UPO.

Furthermore, we presented a simple and scalable method for the sterilization of the yeast cells while maintaining the activity of the immobilized UPOs by simple suspension of the cells in isopropanol for 4 h.

Lastly, we provided storage data for different temperatures and handling procedures for suspended and freeze-dried yeast cells with immobilized UPOs.

The results of this study hopefully help the establishment of YSD as a screening tool for the evaluation of UPO variants and as a cheap immobilization method for UPOs in general. Today, yeast surface display is the UPO immobilization method with the highest activity yield and additionally provides very simple downstream processing and does not require any additional carrier material, which makes it likely the cheapest immobilization method possible (phases 1 and 3–5 of immobilized enzyme processes; [10,12]). With this study, we established efficient cultivation methods for the production of these cheaply immobilized UPOs and identified suitable storage methods as well as a way to address issues of the bioactive nature of the immobilization carrier by sterilization (phases 2 and 6 of an IEP). The main drawbacks of this method remain in the catalysis phase of an immobilized enzyme process (phase 7): the added value of immobilized enzymes for catalytic purposes is in the recovery or retention of the enzyme during repeated or continuous catalysis. Although repeated use has been demonstrated for YSD-UPOs [10], the reaction setup in that study was very simple and resulted in low productivity, and continuous catalysis has yet to be shown. The small particle size of the yeast cells might make this carrier prone to membrane fouling or clogging, so a suitable reactor setup has to be developed to fully harvest the other process-related advantages of YSD-UPOs. With an efficient and scalable catalysis setup in place, the path towards industrial application of UPOs across all process steps would be paved, which could contribute to several of UN’s sustainable development goals like goals 12 and 13 (responsible consumption production and climate action) but also goals 8 and 9 (decent work and economic growth and industry, innovation and infrastructure).

## Data Availability

The original contributions presented in this study are included in the article/Supplementary Materials. Further inquiries can be directed to the corresponding author(s).

## Supplementary Materials

The following supporting information can be downloaded at: https://www.mdpi.com/article/10.3390/bioengineering12080822/s1, Figure S1: Correlation between the backscatter signal measured by a BioLector XT^®^ with the optical density of cultures of *Komagataella phaffii* X33 pPpB1_*Aae*UPO_PaDa-I_SAG1 and *Komagataella phaffii* X33 pPpB1_*Aae*UPO_PaDa-I_sfGfp_SAG1 at different temperatures; Figure S2: Correlation between the Gfp-signal with backscatter signal, both measured by a BioLector XT^®^ of cultures of *Komagataella phaffii* X33 pPpB1_*Aae*UPO_PaDa-I_SAG1 and *Komagataella phaffii* X33 pPpB1_*Aae*UPO_PaDa-I_sfGfp_SAG1 at different temperatures; Figure S3: Correlation between the volumetric ABTS-activity with Gfp-signal, measured by a BioLector XT^®^ of cultures of *Komagataella phaffii* X33 pPpB1_*Aae*UPO_PaDa-I_SAG1 and *Komagataella phaffii* X33 pPpB1_*Aae*UPO_PaDa-I_sfGfp_SAG1 at different temperatures; Figure S4: Correlation between the volumetric ABTS-activity, with Gfp-signal, measured by a BioLector XT^®^ and corrected by the Gfp signal/backscatter signal correlation of cultures of *Komagataella phaffii* X33 pPpB1_*Aae*UPO_PaDa-I_SAG1 and *Komagataella phaffii* X33 pPpB1_*Aae*UPO_PaDa-I_sfGfp_SAG1 at different temperatures; Figure S5: Correlation of the slope and y-axis intercept of graphs shown in Figure S1 with the cultivation temperature; Figure S6: Correlation of the slope and y-axis intercept of graphs shown in Figure S2 with the cultivation temperature; Figure S7: Correlation of the slope and y-axis intercept of graphs shown in Figure S4 with the cultivation temperature; Table S1: Analysis of Variance (ANOVA) for reduced quadratic model of maximally reached volumetric activity; Table S2: Final Equation of maximally reached volumetric activity in Terms of Actual Factors; Table S3: Analysis of Variance (ANOVA) for reduced quadratic model of maximally reached volumetric activity per OD_600_; Table S4: Final Equation of maximally reached volumetric activity per OD_600_ in Terms of Actual Factors; Table S5: Confirmation run evaluation for the models for maximally reached volumetric activity and maximally reached volumetric activity per OD_600_; Figure S8: Effects of different storage conditions on YSD-UPOs.

## Abbreviations

The following abbreviations are used in this manuscript:

ABTS	2,2′-azino-bis(3ethylbenzothiazoline-6-sulfonic acid)
BMGY	Buffered medium with glycerol and yeast extract
BMMY	Buffered medium with methanol and yeast extract
DO	Dissolved oxygen
IEP	Immobilized enzyme process
KPI	Key performance indicators
MeOH	Methanol
OD600	Optical density at 600 nm
Pi	Phosphate
PTM1	Pichia Trace Metal solution 1
RAE	Recovered activity efficiency
RT	Room temperature (≈20 °C)
SDG	Sustainable development goals
sfGfp	Superfolder Green fluorescent protein
STA	Space–time activity
STR	Stirred tank reactor
UN	United Nations
UPO	Unspecific peroxygenase
YPD	Yeast-Peptone-Dextrose (medium)
YSD	Yeast surface display
