# Supplementary material for: Optimizing Yeast Surface-Displayed Unspecific Peroxygenase Production for Sustainable Biocatalysis
Source: Bioengineering (Basel). 2025 Jul 30;12(8):822. doi: 10.3390/bioengineering12080822 (PMC12383419; doi:10.3390/bioengineering12080822)
Supplement: Supplementary file 1 [file bioengineering-12-00822-s001.zip › bioengineering-3775228-supplementary.pdf]

bioengineering

Supporting Information

Optimizing Yeast Surface Displayed UPO Production from Screening to Lab Scale

Niklas Teetz[1], Luc Zuhse[1] and Dirk Holtmann[1,*]

[1] Process Engineering in Life Sciences 2 – Electro Biotechnology, Karlsruhe Institute of Technology, Fritz-Haber-Weg 4, 76131 Karlsruhe, Germany; dirk.holtmann@kit.edu
* Correspondence: dirk.holtmann@kit.edu

1. Supporting Results

1.1 Correlating Gfp-signal to volumetric activity

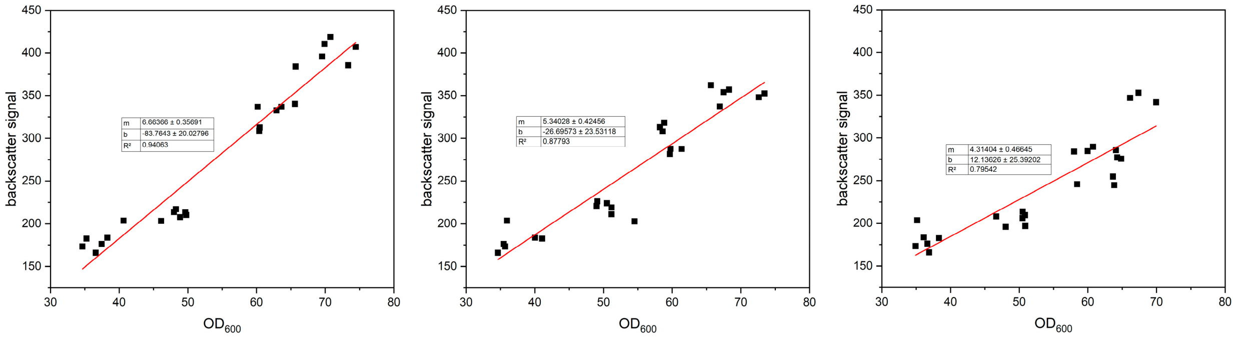

(**a**) (**b**) (**c**)

Figure S1. Correlation between the backscatter signal measured by a BioLector XT® (gain=1) with the optical density of cultures of *Komagataella phaffii* X33 pPpB1_*Aae*UPO_PaDa-I_SAG1 and *Komagataella phaffii* X33 pPpB1_*Aae*UPO_PaDa-I_sfGfp_SAG1 at different temperatures: (**a**) at 22°C (**b**) at 27°C (**c**) at 32°C.

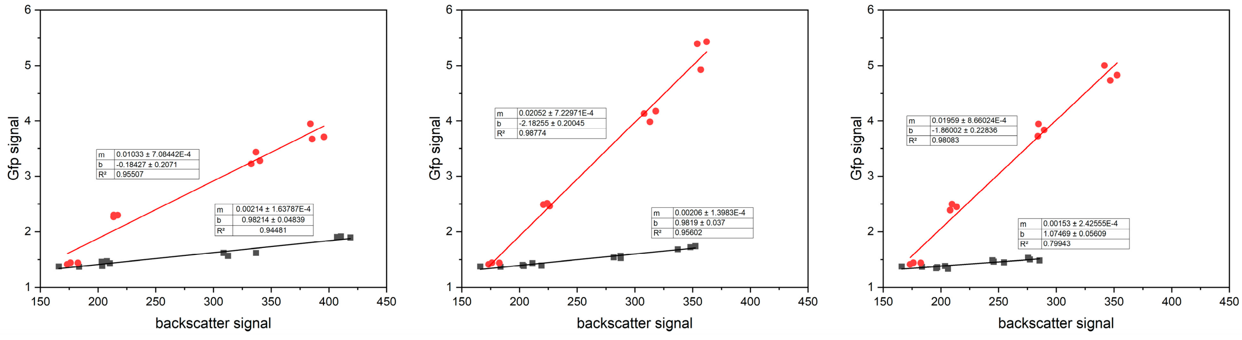

(**a**) (**b**) (**c**)

Figure S2. Correlation between the Gfp-signal with backscatter signal (gain = 1), both measured by a BioLector XT® of cultures of *Komagataella phaffii* X33 pPpB1_*Aae*UPO_PaDa-I_SAG1 (black) and *Komagataella phaffii* X33 pPpB1_*Aae*UPO_PaDa-I_sfGfp_SAG1 (red) at different temperatures: (**a**) at 22°C (**b**) at 27°C (**c**) at 32°C.

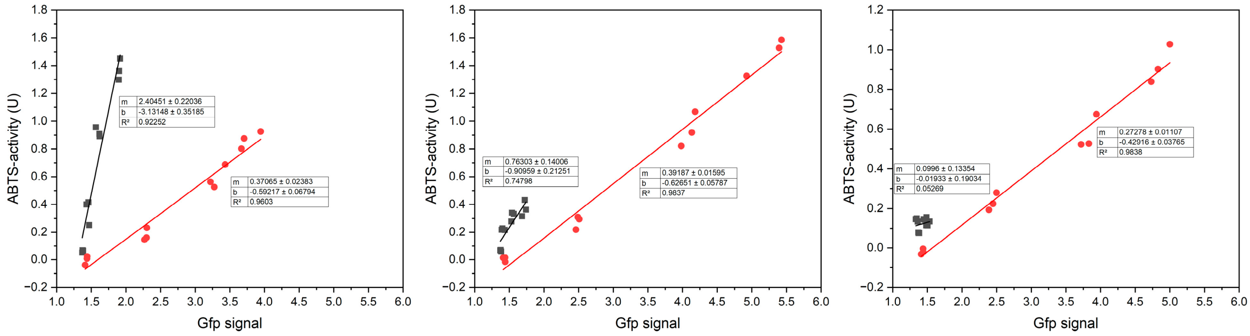

(**a**) (**b**) (**c**)

Figure S3. Correlation between the volumetric ABTS-activity with Gfp-signal, measured by a BioLector XT® of cultures of *Komagataella phaffii* X33 pPpB1_*Aae*UPO_PaDa-I_SAG1 (black) and *Komagataella phaffii* X33 pPpB1_*Aae*UPO_PaDa-I_sfGfp_SAG1 (red) at different temperatures: (**a**) at 22°C (**b**) at 27°C (**c**) at 32°C.

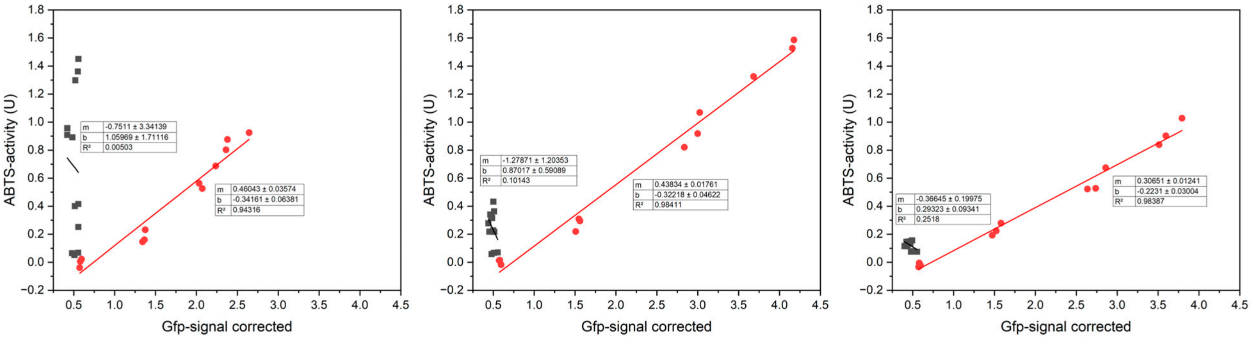

(**a**) (**b**) (**c**)

Figure S4. Correlation between the volumetric ABTS-activity, with Gfp-signal, measured by a BioLector XT® and corrected by the Gfp signal/backscatter signal correlation of cultures of *Komagataella phaffii* X33 pPpB1_*Aae*UPO_PaDa-I_SAG1 (black) and *Komagataella phaffii* X33 pPpB1_*Aae*UPO_PaDa-I_sfGfp_SAG1 (red) at different temperatures: (**a**) at 22°C (**b**) at 27°C (**c**) at 32°C.

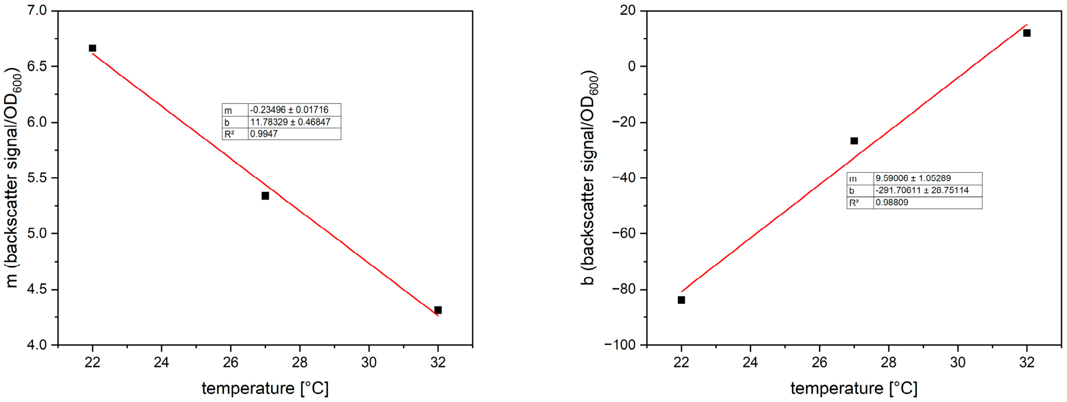

(**a**) (**b**)

Figure S5. Correlation of the slope (**a**) and y-axis intercept (**b**) of graphs shown in Fig. SI 1 with the cultivation temperature.

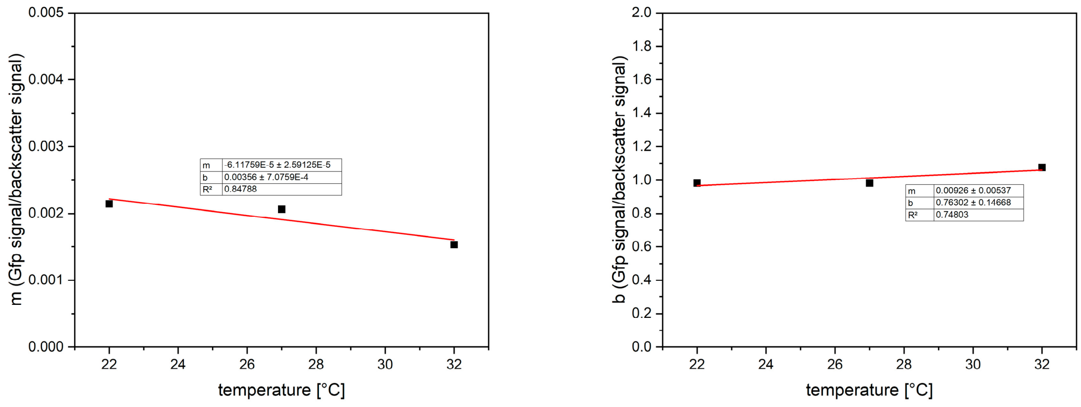

(**a**) (**b**)

Figure S6. Correlation of the slope (**a**) and y-axis intercept (**b**) of graphs shown in Fig. SI 2 (red data points + linear regression) with the cultivation temperature.

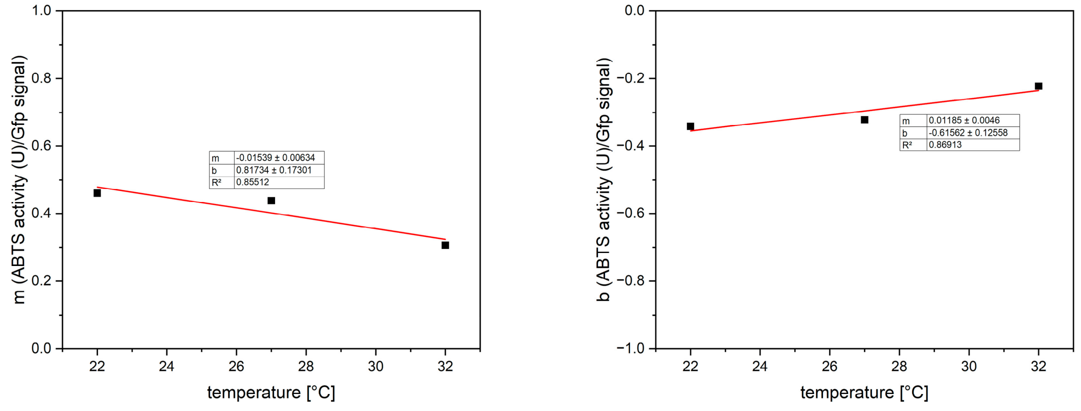

(**a**) (**b**)

Figure S7. Correlation of the slope (**a**) and y-axis intercept (**b**) of graphs shown in Fig. SI 4 (red data points + linear regression) with the cultivation temperature.

1.2 Modelling of activity dependent on cultivation parameters in a BioLector XT®

Table S1. Analysis of Variance (ANOVA) for reduced quadratic model of maximally reached volumetric activity. The Model F-value of 16.91 implies the model is significant. There is only a 0.01% chance that an F-value this large could occur due to noise. P-values less than 0.0500 indicate model terms are significant. In this case A, AD, B², D² are significant model terms. Values greater than 0.1000 indicate the model terms are not significant. The insignificant terms B and D are kept to maintain model hierarchy because B² and D² are significant. The Lack of Fit F-value of 1.22 implies the Lack of Fit is not significant relative to the pure error. There is a 40.06% chance that a Lack of Fit F-value this large could occur due to noise.

Source	Sum of Squares	df	Mean Square	F-value	p-value	
**Model**	1.87	7	0.2670	16.91	< 0.0001	significant
A-Glyc%	0.8516	1	0.8516	53.94	< 0.0001	
B-ODinoc	0.0001	1	0.0001	0.0064	0.9372	
D-Temp	0.0041	1	0.0041	0.2616	0.6146	
AD	0.3125	1	0.3125	19.80	0.0002	
A^2	0.0547	1	0.0547	3.47	0.0774	

B^2	0.1327	1	0.1327	8.40	0.0089	
D^2	0.6471	1	0.6471	40.99	< 0.0001	
**Residual**	0.3157	20	0.0158			
Lack of Fit	0.2040	12	0.0170	1.22	0.4006	not significant
Pure Error	0.1117	8	0.0140			
**Cor Total**	2.18	27				

Table S2. Final Equation of maximally reached volumetric activity in Terms of Actual Factors.

**max. volumetric activity**	**=**
-10.45104	
+0.884462	A-Glyc%
-0.150141	B-ODinoc
+0.792726	D-Temp
-0.035303	AD
-0.060176	A^2
+0.029233	B^2
-0.013786	D^2

Table S3. Analysis of Variance (ANOVA) for reduced quadratic model of maximally reached volumetric activity per OD_{600}. A Square Root transformation was applied. The Model F-value of 22.20 implies the model is significant. There is only a 0.01% chance that an F-value this large could occur due to noise. P-values less than 0.0500 indicate model terms are significant. In this case A, B, AC, AD, BC, B^2, D^2 are significant model terms. Values greater than 0.1000 indicate the model terms are not significant. The insignificant terms C and D are kept to maintain model hierarchy because AC, AD, BC and D^2 are significant. The Lack of Fit F-value of 0.75 implies the Lack of Fit is not significant relative to the pure error. There is a 66.94% chance that a Lack of Fit F-value this large could occur due to noise.

**Source**	**Sum of Squares**	**df**	**Mean Square**	**F-value**	**p-value**	
**Model**	0.0443	9	0.0049	22.20	< 0.0001	significant
A-Glyc%	0.0254	1	0.0254	114.31	< 0.0001	
B-ODinoc	0.0011	1	0.0011	4.85	0.0409	
C-Feedvol	0.0005	1	0.0005	2.44	0.1355	
D-Temp	0.0001	1	0.0001	0.2852	0.5998	
AC	0.0016	1	0.0016	7.18	0.0153	
AD	0.0158	1	0.0158	70.99	< 0.0001	
BC	0.0013	1	0.0013	5.74	0.0277	
B^2	0.0021	1	0.0021	9.40	0.0067	
D^2	0.0079	1	0.0079	35.52	< 0.0001	
**Residual**	0.0040	18	0.0002			
Lack of Fit	0.0019	10	0.0002	0.7529	0.6694	not significant
Pure Error	0.0021	8	0.0003			
**Cor Total**	0.0483	27				

Table S4. Final Equation of maximally reached volumetric activity per OD_{600} in Terms of Actual Factors.

**Sqrt(max. vol. activity/OD_{600})**	**=**
-1.35400	
+0.134787	A-Glyc%

-0.016233	B-ODinoc
+0.000475	C-Feedvol
+0.100837	D-Temp
+0.000691	AC
-0.006585	AD
-0.000393	BC
+0.003565	B^2
-0.001693	D^2

Table S5. Confirmation run evaluation for the models for maximally reached volumetric activity and maximally reached volumetric activity per OD_{600}. Confirmation was done for two points in the model space (Goal 1 & 2).

Goal	Analysis	Predicted Mean	Predicted Median	Std Dev	n	SE Pred	95% PI low	Data Mean	95% PI high
1	max. volumetric activity	0.927253	0.927253	0.125644	6	0.0891033	0.741386	1.07413	1.11312
1	max. vol. activity/OD_{600}	0.0214102	0.0211883	0.00434811	6	N/A	0.0142013	0.0198109	0.0295684
2	max. volumetric activity	0.788008	0.788008	0.125644	4	0.0890239	0.602307	0.808326	0.973708
2	max. vol. activity/OD_{600}	0.0102689	0.010047	0.00300277	4	N/A	0.00599764	0.00697255	0.0151352

1.3 Storage of yeast surface displayed UPOs

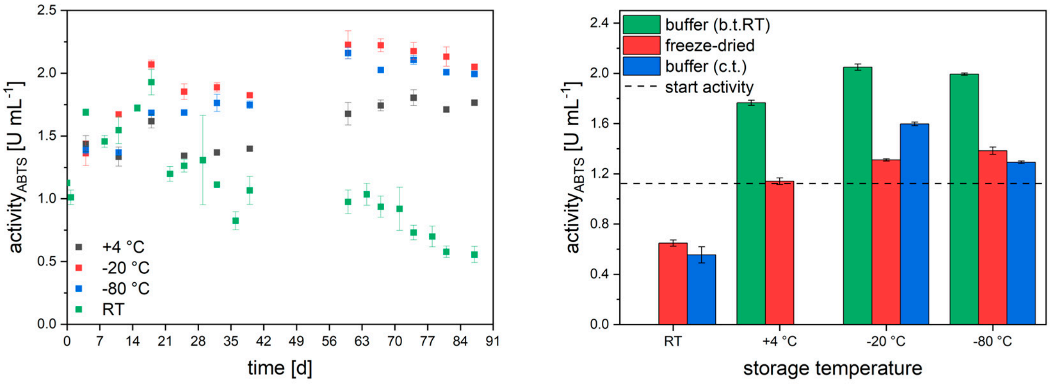

(**a**) (**b**)

Figure S8. Effects of different storage conditions on YSD-UPOs. (**a**) activity development over 87 d for storage at RT, 4°C. -20°C and -80°C in 100 mM KP_i pH = 7. Samples were brought to RT 5 times per week and measured once per week. Data is plotted as average with standard deviation (n = 3). (**b**) Final activity after 87 d of storage for conditions as decribed in (a) (green; in buffer; brought to RT), dry storage of freeze dried YSD-UPOs (red) and storage in buffer at constant temperatures (blue). The dotted line marks the start activity. Data is plotted as average with standard deviation (n = 3).
